# Multi-Omics Strategies for Decoding Smoke-Assisted Germination Pathways and Seed Vigour

**DOI:** 10.3390/ijms21207512

**Published:** 2020-10-12

**Authors:** Utpal Bose, Angéla Juhász, James A. Broadbent, Setsuko Komatsu, Michelle L. Colgrave

**Affiliations:** 1 CSIRO Agriculture and Food, 306 Carmody Rd, St Lucia, QLD 4067, Australia; utpal.bose@csiro.au (U.B.); james.broadbent@csiro.au (J.A.B.); 2 Australian Research Council Centre of Excellence for Innovations in Peptide and Protein Science, School of Science, Edith Cowan University, Joondalup, WA 6027, Australia; a.juhasz@ecu.edu.au; 3 Department of Environmental and Food Sciences, Fukui University of Technology, Fukui 910-8505, Japan

**Keywords:** seed germination, dormancy, vigour, smoke, karrikin, proteomics, multi-omics

## Abstract

The success of seed germination and the successful establishment of seedlings across diverse environmental conditions depends on seed vigour, which is of both economic and ecologic importance. The smoke-derived exogenous compound karrikins (KARs) and the endogenous plant hormone strigolactone (SL) are two classes of butanolide-containing molecules that follow highly similar signalling pathways to control diverse biological activities in plants. Unravelling the precise mode-of-action of these two classes of molecules in model species has been a key research objective. However, the specific and dynamic expression of biomolecules upon stimulation by these signalling molecules remains largely unknown. Genomic and post-genomic profiling approaches have enabled mining and association studies across the vast genetic diversity and phenotypic plasticity. Here, we review the background of smoke-assisted germination and vigour and the current knowledge of how plants perceive KAR and SL signalling and initiate the crosstalk with the germination-associated hormone pathways. The recent advancement of ‘multi-omics’ applications are discussed in the context of KAR signalling and with relevance to their adoption for superior agronomic trait development. The remaining challenges and future opportunities for integrating multi-omics datasets associated with their application in KAR-dependent seed germination and abiotic stress tolerance are also discussed.

## 1. Introduction

Dormancy breakdown and seedling establishments are critical steps of the plant life cycle and are simultaneously controlled by multiple external cues, for instance light, temperature, minerals, water, and nutrients. Smoke produced from the burning or charring of plant materials can stimulate seed germination and improve seed vigour, i.e., rapid and uniform emergence in a range of field conditions; and provide ways to mitigate and overcome abiotic stresses in the field [1,2,3,4]. The clearing of ground cover by wildfires assists the light and water to enter the soil more easily and creates an opportunity for seed germination and the establishment for many species whose germination commences upon sensing smoke or generated heat.

Chemical stimulants present in the char and smoke from fires promote seed germination and seedling establishment. Chemicals such as nitrogen oxides (NO_x_) and cyanohydrins, when present in the smoke or aqueous smoke solutions, have shown a stimulating effect on germination [5,6]. Among the thousands of compounds present in the smoke, the butanolide moiety containing molecule 3-methyl-2H-furo-[2,3-*c*]pyran-2-one and its derivatives, commonly known as karrikin (KAR) [7,8], has shown the highest potential to promote seed germination and assist seedlings with abiotic stress. KARs have been shown to interact with plant phytohormones and currently unknown transcription factor (TF)-like proteins to process endogenous signals for dormancy breakdown and seedling vigour process [9,10,11]. To understand the smoke-assisted regulatory pathways in model and non-model species, large scale genomic screening studies along with targeted experiments have been conducted [12,13,14,15]. These studies were often designed to generate and analyse each dataset in isolation—ignoring the possible interactions between the underlying biomolecules—resulting in findings independent of one another. The investigation and integration of multi-omics datasets are essential to fully understand the complex nature of this enigma. In this review, we first outline the current knowledge about seed dormancy, germination, and vigour; and explain how these stages can be affected with the application of smokes produced from burning plant materials. Then, the interconnection between KARs, strigolactones (SLs) and other phytohormones will be discussed in terms of their role in seed germination and abiotic stress management. Finally, the application of proteomics and multi-omics strategies will be discussed for agronomically important crop species to demonstrate how different ‘omics’ data can be investigated to explore the crosstalk between different pathways and abiotic stress tolerance in crop species. As an example, a protein–protein interaction map will be built using proteins related to KAR and SL-associated Gene Ontology (GO) terms in *Arabidopsis thaliana*. Then, gene expression patterns of homologous genes associated with KAR and SL-metabolism or signalling GO terms in wheat will be analysed using the transcriptome data of different plant development stages and abiotic and biotic stress conditions, which lay the foundation for future experiments to explore their application potential in crops.

## 2. Seed Dormancy/Germination and Environmental Factors that Affect Seed Germination

### 2.1. Seed Dormancy, Germination, and Vigour

Seed dormancy is a static state where the seed can halt germination until favourable conditions are present. This state of dormancy is maintained in various ways across the plant kingdom to ensure the establishment of a new plant generation [16,17]. The dormant seed is unable to germinate within a given period under the effect of any abiotic factors such as temperature, light, water, etc. The seed dormancy mechanisms are interconnected with the plant genetic systems and the abiotic environment, both required to initiate the germination process. Dormancy is not just defined as the characteristics of the seed that is unable to germinate but also as an inherent trait of the seed that determines the conditions required for germination [18,19]. Seed dormancy can be classified into five sub-classes: physiological, morphological, morphophysiological, physical and combinatorial dormancy [20]. 

Seed germination is a complex process where the seed undergoes several changes including: recovery from maturation drying; starting metabolic operations; completing critical cellular events that allow the embryo to emerge; and preparing for seedling growth [21,22,23]. This process spans from water uptake (imbibition) by the seeds to the appearance of the embryo, throughout the surrounding structures. Germination begins with the imbibition of water by the dry seed (Phase I) until the matrices and cell contents are adequately hydrated. Then, the seeds uptake a limited amount of water (Phase II), followed by the increase re-uptake of water (Phase III) eventually completing the germination cycle. Overall, the mature seed must rapidly shift from a desiccated state to a germination-dependent development program for seedling growth [22]. 

Seed vigour is an integral seed feature that determines its potential for rapid uniform germination success and subsequent development across various environmental conditions. Seed germination and seedling emergence is controlled by a network of molecular mechanisms that are cumulatively defined as seed vigour. Experiments using ‘omics-based’ approaches have found multiple factors including the quality of messenger RNAs stored during the seed maturation stage, proteostasis, DNA integrity, cell metabolism and connection with hormonal pathways, to all play a key role in seed vigour [22]. The loss of seed vigour is related to the reduction of the ability of seeds to carry out all the physiological functions during germination. Physiological ageing causes the reduction of seed vigour, which includes changes in cell membrane integrity, enzyme activity and protein synthesis. Seed vigour is not a single factor but a concept associated with multiple factors: the rate and uniformity of seed germination and seedling growth; ability to receive the environmental signals of favourable conditions; storage conditions; and performance during the storage conditions, in particular to retain their ability to germinate [24]. Proteomics and bioinformatic studies have found that the activation of pathways such as sulphur amino acid, lipid and starch metabolism, protein synthesis (increase the abundance of translation initiation factors) and abscisic acid (ABA) signalling pathways correlate with increased seed vigour [25]. Although studies have tried to establish a seed vigour mechanism, the interaction between genetic, DNA repair mechanisms, storage conditions and environmental factors with these biochemical changes are not fully understood. 

### 2.2. Environmental Factors that Affect Seed Germination

Seeds start to breakdown the dormancy state and activate the germination pathways upon receiving cues from surrounding environments. The surrounding environment and the physical soil characteristics help to establish their microclimate conditions. Various mechanisms are involved in the breakdown of dormancy. For instance, after ripening or chilling, dormancy breakdown depends upon whether the seeds are dry or wet. Once dormancy starts to breakdown, the seeds respond to the cues from the microenvironment and sense the overall environment. Light, temperature, soil moisture, nutrients and chemical cues, i.e., smoke, determine the rate and extent of germination of non-dormant seeds [26]. Comprehensive reviews have been published on how various biotic and abiotic factors influence the seed germination process [16,18,21,22,23,24]. The role of chemical cues, i.e., the smoke-assisted germination process will be discussed in later sections.

## 3. Role of Smoke, Smoke Generation, and Components

### 3.1. Role of Smoke in Germination 

Wildfire plays a crucial role in ecosystems to facilitate the germination process for many plant species around the world. Plants have evolved with the fire-dependent reproductive strategies over time to build natural ecosystems. Smoke generated from burning plant materials enhances seed germination in over 1200 phylogenetically diverse plant species [27,28,29,30]. Studies have shown the impact of smoke originating from wildfire to restore the vegetation of fire-prone native species in the Mediterranean region [31]; Australia [32]; South Africa [33]; California [34]; and, the Mediterranean basin [35]. Additionally, its positive impact on the germination of cereal crops [2,36]; weed species [1]; horticultural crops [14]; and medicinal plants [37] has also been confirmed. The smoke-dependent germination process improves germination success across phylogenetically diverse plant groups including gymnosperms; angiosperms [33]; annuals; bi-annuals; short-lived species; and, perennial herbs [38], in different weather regions around the globe from tropical to the fire-prone Mediterranean region, disturbed habitats, representing a broad array of fire-prone environments [30,32]. Smoke has been used as an effective mediator to release dormancy in the laboratory [39] and field conditions [14]. Altogether, the germination response to smoke is not just associated with plant traits such as habitat requirements, seed bank, type, life form, and seed morphology, but also the chemical stimuli that alleviate the dormancy state.

Seeds receive natural cues from the environment that help them to respond to the physical (temperature and light) and chemical (smoke, gas, nutrients) conditions as germination cues are associated with fire [29,30]. For example, the heat produced from the fire can help to breakdown or desiccate the seed coat [40] or may reduce the expression of dormancy controlling hormone abscisic acid (ABA)-related genes during post-germination events [41]. Hard-coated seeds with a prominent waxy cuticle and dense palisade layer of sclereids enforce dormancy by making the seed coat impermeable to water. Brief heat shock between 80 and 120 °C is enough to cause the seed to imbibe water by loosening cells or by denaturing germination inhibitors. For some species, heat shock alone may work, but some heat-induced species also require light and/or cold stratification [9,42]. Heat and the chemical constituents generated from the fire events may also stimulate the embryo growth by inducing somatic embryogenesis during seed germination [39]. The application of smoke has been shown to activate the KAR receptors in Arabidopsis [43], presenting the question as to whether smoke can be applied to crop species to develop drought-tolerant plants.

### 3.2. Smoke Generation and Application 

Smoke water produced by the combustion of vegetation has been widely used to increase germination and seedling vigour due to its convenient mode of application. The chemical constituents present in the smoke readily dissolve in water and the dilution of this smoke water has been shown to markedly improve germination rates [1,7,44] and abiotic stress tolerance [3,45,46]. In this technique, smoke was generated in a drum, and using compressed air, was bubbled through distilled water [33]. A wide range of plant materials has been used to generate aqueous smoke extracts using this technique [1,29,30,47]. Studies have shown that all plant materials produce similar chemical compositions and can be applied in diluted forms ranging from 1:10 to 1:100, *v*/*v* [48] or 1000–2000 ppm [2] to enhance germination events. However, a recent study has shown that the dilution ratio of smoke water and distilled water 1:2500 (*v*/*v*) from the sub-dilution 25:75 (*v*/*v*) of smoke water was the best for the germination of lettuce seeds in the dark, achieving a 91% germination rate against the water control that had only 7–10% germination [47]. Although these dilution ranges have been found to be effective to promote the germination events, the response varies widely across plant species. 

### 3.3. Active Components in Smoke

Chemical compounds present in the smoke stimulate germination and enhance seedling vigour in several agricultural, horticultural and wild plant species [24,30]. Smoke contains thousands of compounds including organic acids, organic bases, alcohols, aldehydes, esters, alkyl aryl ethers, furan and pyran derivatives, ketones and diketones, lactones, phenolic derivatives, guaiacol derivatives, syringol derivatives, hydrocarbons, and nitrogenated derivatives [29,32,49,50,51]. De Lange and Boucher (1990) demonstrated that active component(s) of gas phase smoke are water-soluble and that a smoke-saturated solution is effective in improving seed germination [33]. Since then bioactive compounds having either stimulatory or inhibitory activity on seed germination have been identified in smoke [47,52]. Both organic and inorganic chemicals originating or released from smoke help stimulate the germination of seeds. Nitric oxide was discovered as an active component in the smoke that can stimulate the maximum level of germination in the California chaparral annual *Emmenanthe penduliflora* [53]. Dormant seeds from this species germinate fully upon brief exposure to smoke or vapor generated from smoke-treated sand or paper, and the germination capacity can be comparable with 500 ppm NO_2_ [53]. Chaparral wildfires generate enough NO_2_ from the combustion of organic matter or from postfire nitrification, which helps trigger germination events in a process independent from imbibition. Notably, all pyroendemics (seedling germination and successful seedling recruitment for plant varieties that depend on postfire environments) in chaparral showed a significant response to this gas and thus it appears that other chemicals may also act as germination stimulants in smoke and charred wood [54]. In addition, other nitrogenous compounds generated from combustion include cyanohydrin (glyceronitrile), which can be slowly converted to cyanide and stimulate seed germination in a wide range of fire-responsive species from different continents [6]. 

Of the thousands of different compounds in smoke, some of them act as enhancers while some inhibit the germination process [55,56]. Dose-dependent responses of smoke compounds have also been demonstrated [49]. Studies have been conducted in recent decades to identify the active constituents from smoke [28,53,57]. A compound was purified as 3-methyl-2H-furo-[2,3-*c*] pyran-2-one from the burning of cellulose- and plant-derived smoke material by using bioassay-guided fractionation [7,8]. Later, this compound was named as karrikinolide (or KAR_1_ or KAR) after the Noongar aboriginal word for smoke “Karrik” [50]. KARs are produced by the pyrolysis of cellulose and sugars—thought to be deposited on the soil surface during a fire, and eventually absorbed by the seeds buried in the soil over the time after being dissolved by rain. To date, six different isoforms of the KAR family (KAR_1_–KAR_6_) have been reported; all of them contain a five-membered butanolide ring and a six-membered pyran ring [27,50]. The main difference among the KAR family members is the number and location of methyl group(s) [50]. KAR_1_ is the most abundant and bioactive within smoke [50]. Notably, KARs share structural similarities with plant hormone SLs—both belong to the butanolide class and trigger germination but originate from different sources [10].

The effect of KARs is not limited to the fire-prone plant species but improves seedling vigour, root development and abiotic stress tolerance in many crop species [35,42,43]. In Arabidopsis, KARs have been found to enhance germination and seedling responses to light [58]. Variable responses to KARs in terms of germination are observed to be dependent on plant species, their origin (geographical location) and/or seed maturity stage. For example, germination is delayed upon KAR exposure in soybean by increasing ABA synthesis and impairing GA biogenesis [59]. Additionally, the germination of other species such as *Capsella bursapastoris*, *Bromus sterilis* and *Alopecurus myosuroide* are also delayed by KARs [9]. The ubiquitous nature of KAR-induced germination success indicates that it is an ecologically significant germination cue [58]. However, interestingly, the activity of KAR is also dependent on light intensity, the duration that seeds spend in dormancy and the surrounding ecological conditions [60].

KAR_1_ has the highest water solubility, low volatility and is the most abundant compound among the other subtypes in smoke residue [51]. Studies have reported that KAR_1_ can be produced by burning pure xylose, glucose, or cellulose, and it has been proposed that KAR_1_ is derived from a pyranose sugar [6]. KAR_1_ produced during combustion condenses and is deposited as the smoke cools and remains close to the fire-affected areas [4]. Karrikin is deposited along with the nutrients present in the ash. The removal of litter containing allelopathic chemicals and changes in light quality or accessibility affects the rate of germination in the fire-affected sites, but not in the unburned areas in the rainy months [27]. Overall, the seeds’ response to KARs can provide an inherent capacity for plants co-located with annual fire events to enhance their ability and potency in the post-fire landscape.

## 4. Karrikins and Strigolactones Share the Common Signalling Pathways in Plants

Smoke-derived KARs are a class of butanolide molecules that can mimic the endogenous signalling molecules (karrikin-insensitive 2 (KAI2)-ligand, KL) and stimulate the seed germination and growth of many species; control the photomorphogenic development of seedlings, leaf shape, root growth and root hair development [4,58]. Plants respond to KARs, which trigger the signalling pathways in a closely related manner to plant phytohormone SLs; these hormones are carotenoid-derived phytohormones with butanolide moieties [61,62]. A recent study highlighted the existence of at least two butanolide signalling pathways in vascular plants [63] where different members of the D14 α/β hydrolase protein family are essential for SL/butanolide signalling. The butanolide ligand, i.e., KL, responsible for the early KAI2 signalling pathway, is still unknown [15,42], and is neither KAR or SL. KAI2 has gone through a gene duplication event, which resulted in D14 clade during the time of evolution [64]. Notably, the D14 pathway perceives the canonical SL ligand and diverged from the KAI2 clade, both evolutionary and physiologically, in terms of distinct plant growth regulators [65]. Unlike KARs, SLs are known to be synthesized in plants and can regulate growth at various developmental stages, including shoot branching [66]; root development [67]; nodule formation [68]; and the control the strategic resource allocation in response to environmental changes [69]. 

The perception of KARs is similar to SLs in plants where both hormones bind to the related α/β-fold hydrolases such as Dwarf14/decreased apical dominance2 (DAD2) in rice, and KAI2 in Arabidopsis [11]. The third member of the family in Arabidopsis, DWARF 14-like2 (DLK2) is structurally very similar to D14 and KAI2, and its potential role in hypocotyl elongation has recently been described [63]. The main subtype of KARs—KAR_1_—can stimulate germination regulatory pathways while the smoke compound trimethylbutanolide (TMB) can inhibit KAR_1_ activities [55]. Dose–response analysis followed by transcriptome measurement revealed that KAR_1_ and TMB are not competitors; TMB regulates germination in a concentration-dependent manner. In KAR_1_-sensitive lettuce, TMB helps to upregulate the genes associated with the ABA pathway while KAR_1_ suppresses the dormancy-related genes [55]. Both SL and KAR interact with the F-box protein More Auxiliary Branches (MAX2) for ubiquitylation and the downstream degradation of KL-or SL-signalling proteins of the suppressor of MAX-like (SMXL) family of transcription repressor [11,12], while DLK2 acts independently of MAX2 [63]; however, its transcriptional regulation is accomplished through MAX2.

The Arabidopsis MAX2 encodes an F-box protein with a C-terminal leucine-rich repeat containing domain (LRR) similar to TIR_1_ and COI_1_, which are auxin and jasmonic acid receptors, respectively. In Arabidopsis and rice, MAX2 interacts with SKP1 and SCF (Skp-Cullin-F-box) ubiquitin ligase, and eventually acts in proteolytic targeting [11]. The D14 gene product, which is also part of α/β-fold hydrolase superfamily, is naturally expected to assume the role of SL receptor that directly perceives SL and acts as gibberellin receptor gibberellin insensitive dwarf1 (GID1) [70]. The interactions between D14 and MAX2 are facilitated by the GR_24_ [71], suggesting an SL-dependent formation of the SCF E3 ligase complex containing D14. The SCF E3 ligase complex with GID1 in GA signalling is the analogous formation, where GID1 functions as an adaptor protein of the GA-dependent recognition of substrate DELLA proteins. Studies have shown that the substrate of the SCF^D^_3_/^MAX^_2_^/D^_14_ E3 ligase complex is SMXL_1_ [72], which acts as a signalling repressor upon being treated with GR_24_. Although SMXL protein families show sequence homology and signalling repressor function similarity with an ATPase protein of the ClpB/Heat Shock Protein 100 (HSP100) class, the molecular function of these proteins remains unknown [73]. 

In rice and Arabidopsis, the KAR receptors are characterised as D14 homolog D14-like (D14L) and D14 paralog KAI2, respectively [74]. D14 and D14L/KAI2 share a relatively high amino acid sequence identity (~50%) [74]. In the KAR signalling pathway, D3/MAX2 is common, but the substrate proteins are SMAX1 and SMAX2 [75]. Although the SL and KAR signalling pathways involve MAX2, the divergent growth responses to SL and KARs in plants show that they are perceived differently [58,76]. Interestingly, D14 and KAI2 control complementary aspects of MAX-2 regulated growth; for instance, D14 regulates shoot branching in an SL-responsive phenotype by regulating the transcription factor bri1-EMS-suppressor 1 [77]. On the other hand, KAI2 regulates seed germination in a KAR-sensitive phenotype [78]. Both D14 and KAI2 influence root length, leaf morphology and regulate the hypocotyl elongation in seedlings [12,78]. Overall, the questions remain as to how KARs facilitate the formation of the KAI2–MAX2–SMXL complex and then activate the ubiquitination and degradation of SMXL proteins.

## 5. Co-Activation of KAR and Plant Innate-Hormonal Pathways

Plant hormones play key roles in the integration of diverse environmental cues with signalling networks and pathways in plants. KAR is considered a plant hormone, though it is not produced in planta, due to its diverse function in seed germination and plant vigour [4]. Gibberellic acid (GA) helps to breakdown dormancy, while ABA and indole-3-acetic acid (IAA) promote dormancy and delay seed germination [20,21]. Recent studies have shown that KARs can regulate seed germination and the seedling establishment process by alleviating shade avoidance through triggering the plant phytochrome signalling pathways such as ABA, GA and IAA [9,79]. For example, ABA antagonistically regulates the KARs’ effects during germination and thus KARs requires the activation of the GA biosynthetic pathways to complete the germination process in Arabidopsis [80]. In Arabidopsis, KARs can promote the expression of GA biosynthesis genes *GA3ox1* and *GA3ox2* during germination [58]. Both ABA and IAA can synergistically control seed dormancy; however, the mechanism of how these two hormones work together to establish the dormant state and the interrelationship between GA and IAA is still unknown. Of interest, KARs can suppress the expression of the auxin-responsive protein *IAA1* in Arabidopsis during germination [11,81]. IAA regulates the shade avoidance response in plants by controlling hypocotyl elongations along with GA and brassinosteroid [9,82]. Given that KARs suppress *IAA1* expression, it may thus be possible that the biosynthesis and transport of IAA during seedling establishment may be inhibited by KARs. However, the precise mechanism of how KARs dominate seedling establishment by the regulation of IAA biosynthesis is unclear. Overall, KARs and IAA interact with ABA during germination, but how the KARs interact with the ABA pathways by regulating the IAA signalling pathway remains unknown.

Hormone receptors in plants perform two major roles: signal perception and signal transfer to downstream molecules. The hormone binding sites of the receptors generally contain hydrophobic pockets with polar patches to trigger specific interactions with the polar groups of the hormones. These hormones are allosteric inducers that induce conformational changes in receptors upon hormone perception to switch on or facilitate the transfer of signals to downstream effector proteins. Hormone-dependent proteolysis of cullin-RING ubiquitin ligase (CRL) complexes play a key role in the signalling pathways of major plant hormones such as IAA, jasmonic acid (JA), GA, SL, KAR, ABA and ethylene [79]. The auxin and JA receptors are F-box proteins with leucine-rich repeat (LRR) domains that recognise the substrate proteins for ubiquitinylation in a hormone-dependent manner. Notably, the GA, SL, and KAR receptors are members of the α/β hydrolase superfamily and act as F-box protein-bound adaptor proteins that recognise substrate proteins for ubiquitylation in a hormone-dependent manner [83]. Receptor GID1 activates upon binding to GA exposing the hydrophobic surfaces of the catalytic His residue of the Ser–His–Asp catalytic triad with a Val/Ile residue that can interact with DELLA proteins [70]. In contrast to GID1, the SL and KAR receptors possess a conserved catalytic triad system. These catalytic residues are essential for the action of SL and KAR receptors, and the SL receptor obviously exhibits catalytic activity with respect to SL hydrolysis, which is essential for SL function [84]. Although the genetic homology-based approach has been used to elucidate the hormonal KAR signalling pathways’ activation through KAI2 and SL signalling, the KAR-induced KAR–KAI2–D3/SMAX1 interactions and MAX2-dependent proteolysis of SMAX1 remain biochemically untested [10]. Additionally, how the SMXL/D53 proteins regulate the germination and growth upon hormone signal perception is currently unknown.

MAX2-dependent KAR signalling pathways have a role in seed germination and development. The MAX2 mutants have shown extended dormancy, epinastic leaves and long hypocotyls under various photomorphogenesis conditions [11,85]. Studies have reported the connection between MAX2 and plant phytohormones, i.e., IAA biosynthesis pathways [9,75]. In Arabidopsis, the expression pattern of MAX genes from SL pathways can modify IAA transportation, and thereby regulate vascular tissue formation and regeneration [86]. This result suggests that IAA and SL can control their levels and distribution in a thorough MAX2 pathway by creating a dynamic feedback loop required for the coordinated control of auxiliary branching. Additional studies have shown that MAX2 can suppress IAA transport through the inhibition of PIN genes responsible for IAA transportation [87]. Furthermore, the ABA and GA biosynthesis pathways are also co-regulated by the MAX2-dependent signalling pathway [88], indicating a shared pathway activation mechanism between the phytohormones and KARs to regulate seed germination and photomorphogenesis. Although the crosstalk between MAX2 and phytohormones has been investigated, the relationship between KAI2 receptors and other photomorphogenesis factors is yet to be understood.

## 6. Application of Proteomics to Understand Smoke Water-Assisted Germination and Abiotic Tolerance in Crops

The advancement of mass spectrometry-based proteomics has enabled the routine measurements of thousands of proteins in terms of their abundance, localisation, interactions, and modifications while connecting the large-scale data to gene expression regulation and function [89,90]. Targeted and untargeted proteomics experiments have been widely used to investigate crop responses to the abiotic stress [89,90,91,92]. Proteomics studies have shown that plant-derived smoke water solutions can improve the abiotic stress tolerance capacity in soybean [3], chickpea [93] and rice [36]. Zhong et al. have shown that treatment with plant-derived smoke can enhance soybean plant growth under flooding stress [94]. A combination of proteomics and metabolomics revealed that smoke water helps to alter arginine metabolism and regulates ornithine synthesis/ubiquitin–proteasome pathways to provide early stage soybean plant development under stress conditions [94]. Interestingly, a recent study has shown that the SL and KAR signalling pathways activate the polyubiquitination and degradation of SMXL2 to regulate hypocotyl elongations in a D14 and KAI2-dependent manner in Arabidopsis [95]. Another proteomics-based study on flooding-affected soybean plants has shown that the treatment with smoke water can increase the accumulation of cell wall-related proteins, sucrose/starch metabolism and glycolysis [3]. Likewise, the application of smoke water has shown to increase the weight and length of flood-stressed soybean root by activating the ascorbate/glutathione pathways [46]. Using proteomics, smoke water-treated chickpea plants showed a perturbation of proteins such as glyceraldehyde-3-phosphate dehydrogenase and fructose-bisphosphate aldolase to support glycolysis pathways in order to enhance seed germination and plant growth rates [93]. Overall, proteomics-based studies have shown great potential to understand the smoke water-assisted germination pathways, which can lead to future research opportunities for agricultural crop improvement.

## 7. Applying Multi-Omics Strategies to Identify Karrikin Receptor Proteins and Establish Their Pathways in Agronomically Important Crops

**Figure 1:**
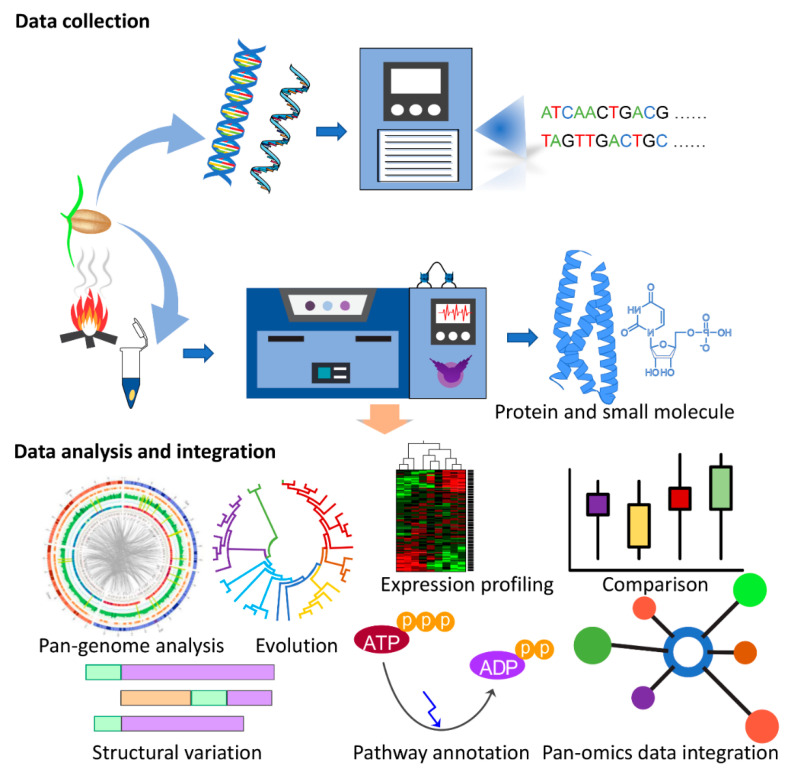
Multi-omics experimental strategy for decoding karrikin (KAR) signalling pathways in crops. The first step is to collect omics data and then analyse and integrate the data in combination to identify the key regulatory mechanisms in plants.

Decoding the genetic basis of agronomic traits is critical for crop improvement. The in-depth knowledge of the underlying genetic variation offers resources for marker development to link genetic variation with agronomic traits via quantitative trait loci (QTL) and genome-wide association studies (GWAS) studies. The availability of reference genome sequencing data from various crop species and their comparison with other species through the comparative pan-genome approach has enabled the understanding of the underlying molecular genetics such as gene function and pathways for biotic and abiotic stress resistance [96,97]. Advancements in next generation sequencing and data processing techniques have improved the understanding of genetic diversity, making it feasible to look beyond the knowledge generated from a model species and explore the evolution of signalling pathways in the specific ecological context of crop breeding programs. Studies have applied comparative genomic approaches to select agronomic traits such as grain yield and quality for rice [98,99], photoperiod sensitivity in maize [100] and grain size and shape in wheat [101]. Despite the wide application of genome sequencing and pan-genome analysis for improving cereal traits, the diversity and role of KAR and SL pathways remain unexplored with regard to their role in shade avoidance, the regulation of germination, seed vigour and plant development across cereal species such as wheat, maize, rice and barley. Notably, many signalling-related experiments were performed on model species such as Arabidopsis. To translate this knowledge from model species to agronomically important crops, the integration of genomics resources with QTL, GWAS, molecular marker and integrated-omics studies can be beneficial and represents a promising strategy for understanding critical insights into the regulation of KAR and SL pathways. The unprecedented availability of reference and pangenome assemblies will facilitate further interdisciplinary research and generate resources to study the combinatorial differences in gene content between crops, supporting the functional characterisation of genes, their evolutionary history and function and facilitate the design of future ‘omics-guided synthetic biology-based experiments to validate their functional importance in crops (Figure 1).

Of the multi-omics approaches, one avenue is to build mutant- or species-specific genomic resources through DNA and RNA sequencing technologies and then use proteomics-guided techniques to validate the gene model and understand the extent of modifications or perturbations at the system level. These proteogenomic models have been developed and widely applied for personalised medical treatment development [102]. Adapting this methodology to plant science also has the potential to answer the remaining questions in KAR signalling that genomic or other ‘omics’ strategies alone have been unable to answer. This will include the investigation of post-translational modification that is critical to signalling and the impact of environmental changes. Specific attention should also be paid towards transcription factors (TFs) as the targeted and dynamic expression of genes, proteins and metabolite biosynthesis and signalling are independently or cooperatively dependent on these in planta [103,104]. To elucidate how TFs influence the KAR-dependent signalling pathways for crop species, additional studies are required to investigate the interaction among TFs and the quantitative responses between DNA, RNA, proteins and the small molecules.

Besides traditional marker selection methods used for breeding, data-driven analyses have been used to establish and identify the molecular strengths (targeting the genome, transcriptome and proteome) of plants, providing a pathway to upregulate their defence-related genes [105] or enzymes [106]. For example, the application of jasmonic acid can induce insect resistance in rice [105]. Likewise, a transmembrane protein BIL4 that regulates cell elongation was found to be affected by the brassinosteroid synthesis inhibitor brasinazole [107]. In a similar way, upon identifying and validating the molecular targets for KAR-dependent seed vigour and/or plant abiotic stress-tolerant pathways for crops, molecular strengthening techniques can be developed by applying exogenous substances that can control or enhance plant growth and development.

**Figure 2:**
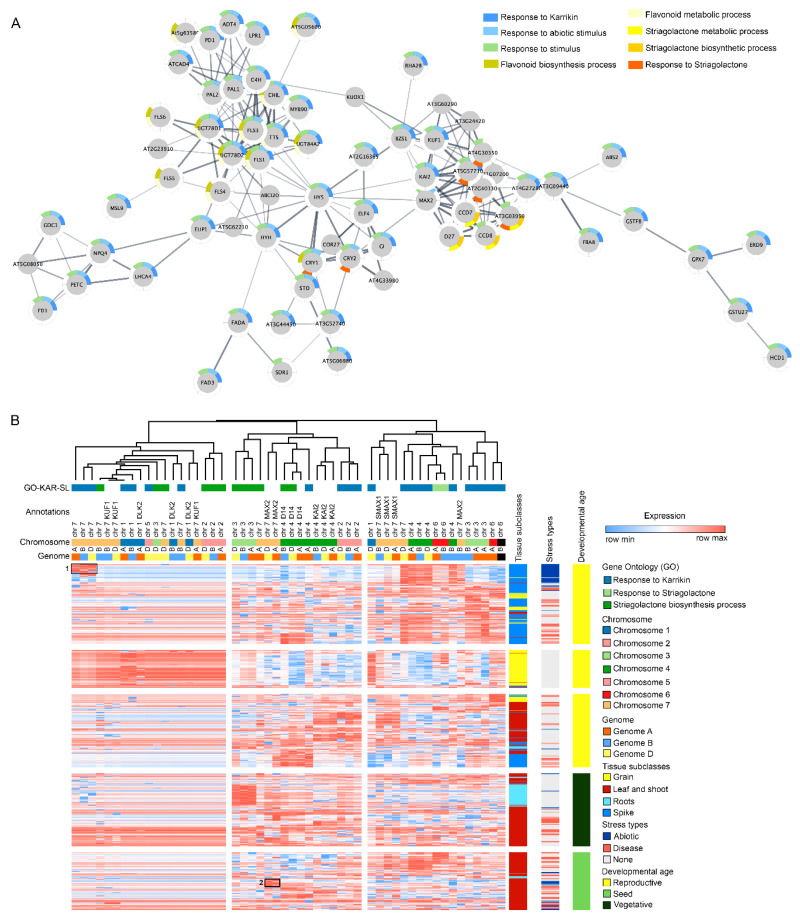
Genes associated with the known KAR and strigolactone (SL) cross talk can be identified in wheat and related crop species. (**A**) The dynamics of selected GO categories for KAR and SL proteins were captured in a protein–protein interaction map using proteins related to KAR and SL-associated GO terms in *A. thaliana*. The network built using the sequences tagged with the KAR and SL gene- associated GO terms provides evidence for the direct relationship between the two hormone signalling pathways. Node colouring in the graph depicts the significantly enriched GO terms for KAR and SL. (**B**) Gene expression patterns of homologous genes associated with KAR and SL-metabolism or signalling GO terms in wheat were analysed using transcriptome data acquired at different plant development stages and abiotic and biotic stress conditions. Dendrograms show the clustering of GO terms associated with KAR and SL, gene annotations, chromosomal locations and the corresponding common bread wheat (hexaploid) genomes A, B and D. The heat map depicts the relative transcript level of KAR- and SL-related gene expression across wheat tissues, stress subtypes and developmental stages. High expression levels in abiotic stress conditions were primarily enriched in chromosome group 7 (marked as 1); MAX2-dependent abiotic stress tolerance and KAR signalling pathways (marked as 2).

To map the tentative orthologous receptor genes for KARs and SLs identified in *A. thaliana*, a genome-wide search was performed using the published genome of agronomically important crops like wheat, barley, rice, maize and sorghum. Gene ontology (GO) terms associated with response to karrikin (GO:0080167), response to strigolactone (GO:190234); strigolactone metabolic process (GO:1901600) and strigolactone biosynthetic process (GO:1901601) were identified from *A. thaliana* and searched against publicly available databases for the aforementioned genomes (Table S1). In Arabidopsis, 133 genes have been associated with KAR response, many of these are present in multiple protein isoforms (Figure 2A). In wheat, 22 genes representing all seven chromosome groups are associated with response to karrikin. In barley, there are 13 genes associated with the GO term. Additionally, 22 genes have been identified in rice, 20 in sorghum and 23 in maize.

To further investigate the wheat gene orthologues identified from interaction networks, the expression data for the gene list (known karrikin response, strigolactone response, strigolactone biosynthesis and the annotated DLK2, MAX2 orthologs and SL function-associated genes) were downloaded from publicly available wheat gene expression databases [108,109]. To understand how KAR and SL play a role in stress response in plants, expression data for samples collected from abiotic or biotic stress experiments were examined. Notably, genes with high expression levels in abiotic stress conditions were primarily enriched in chromosome group 7 (marked as 1; Figure 2B). Likewise, another set of enrichment was observed for genes in the chromosome 4 (Figure 2B).

In Arabidopsis, *KAI2* mutants have shown osmotic stress tolerance by activating DLK2 and Karrikin Upregulated F-BOX1 (KUF1) genes while inhibiting the hypocotyl elongation [13]. Although, a positive correlation has been observed for KAR and SL-related genes with the abiotic stress tolerance, the results herein provide evidence that most of the gene expression was observed in the wheat spike tissue. It is possible that due to the under-representation of stressed root-specific transcriptome data, the identification of any positive correlation with the root and KAR and SL genes was not observed herein. Future transcriptome studies may be performed on wheat roots with or without KAR or SL treatment to understand their roles in abiotic stress tolerance in agronomically important plant species. 

Interestingly, we identified another cluster of genes associated with MAX2-dependent abiotic stress tolerance and KAR signalling pathways (marked as 2; Figure 2B). Studies have shown that KAR and SL play a strong role in plant responses to drought and salt stress through MAX2 pathways in Arabidopsis [43,45]. Here, the interaction network and the clustering analysis have shown that KAR-dependent stress response pathways are active in Arabidopsis. Further exploring the members of the KAR and SL signalling pathways and investigating their orthologues in wheat will enable us to gain a better insight into the molecular mechanisms underlying their roles in crop adaptation to abiotic stress responses. The mapping results presented here should be taken cautiously as these are the tentative leads for KAR and SL pathways in cereal crops. Future follow-up studies should be designed to validate the target genes from cereal crops and elucidate their functional variations in a genome content and structure on crop performance. Extensive genomic profiling with transcriptomics, proteomics and metabolomics across KAR and SL mutants for cereal species would greatly expand the knowledgebase in this area. The results obtained through ‘omics data mining can then provide us with an avenue for developing improved stress-tolerant crop traits.

## 8. Concluding Remarks and Future Perspectives

Comprehensive molecular and structural analyses along with plant phenotype experiments have been performed on SL and KAR signalling pathways in recent decades. However, challenges remain regarding how to translate this new knowledge from model species and apply it to commercial crops. Advancements in acquiring omics data and integrative system biology analyses have enabled researchers to look beyond model plants and explore the activation or regulatory signalling mechanisms in specific ecological contexts. Comparative omics analyses are likely to be the most promising strategies for gaining insights into critical KAR pathway residues, types of regulation and their application feasibility for crops (Figure 1).

**Figure 3:**
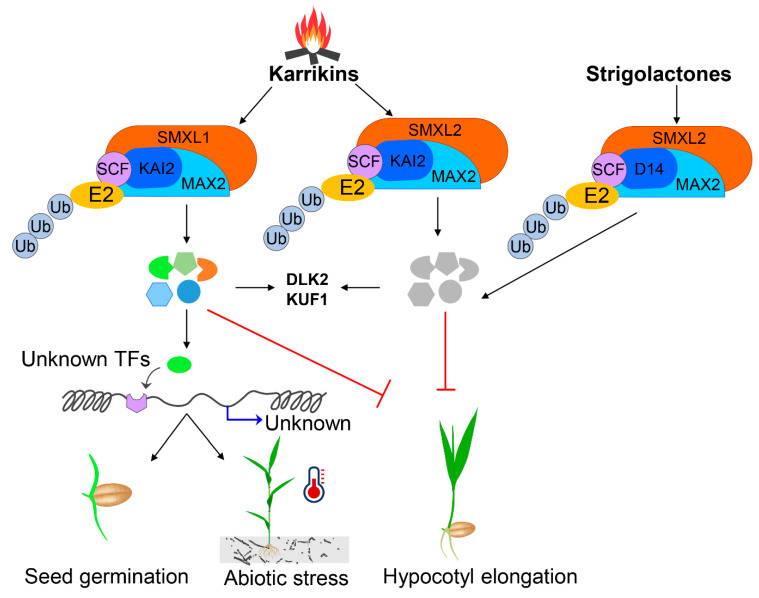
Proposed model of strigolactone and karrikin-assisted germination and abiotic stress tolerance pathways for agricultural crops. The figure has been re-drawn and modified from (Wang et al., 2020; Yao and Waters, 2020; Morffy et al., 2016).

KARs and plant phytohormones such as ABA, GA and auxin can control seed germination and seedling development. However, there are knowledge gaps as to how KARs can interact and precisely synchronise the key signalling pathways during germination, in particular for auxin. The questions remain: why and how different plants species perceive KAR responses (Figure 3); how do different SMXL proteins regulate various downstream signalling responses; besides KAI2 activation, how are KARs metabolised into the active ligand; what is the precise mechanism of action; and, additionally, what is the evolutionary context of the SMXL family that should be considered in order to decode the ligand-receptor activation machineries. 

The advancement of next generation sequencing technologies, mass spectrometry-based proteomics and data mining strategies has enabled the identification and functional characterisation of the genetic factors controlling superior agronomic traits in crops. The integration and application of post-genomic profiling platforms have helped to unravel the complex phenotypes and genetic diversity in plant species [18,110,111]. As we have seen, the response to KARs can be diverse across crop species. Large-scale ‘pan-omics’ approaches can be used as an option to decode the unknown regulatory mechanisms for KAR-dependent pathways. The ‘proteogenomic’ approach, which exploits the information gathered from genomic and proteomic studies, widely used in clinical research [102], can be introduced to explore the KAR and SL-dependent pathways in plants. The pan-genomic and comparative genomics analysis approaches may also be used to identify and screen for the diversity of KAR receptor genes present across different plant species. Comparative -omics and data mining strategies can also be applied to mutants and/or different plant species. Upon the identification of target genes, pathways and the molecular processes for KAR signalling pathways, marker-assisted selection, transgenic, and genome editing approaches may be applied to develop the seeds and plants with enhanced agronomic traits. Multidisciplinary translational research is required to apply these scientific discoveries into useful benefits for crop development and seedling vigour.

## Supplementary Materials

Supplementary materials can be found at https://www.mdpi.com/1422-0067/21/20/7512/s1. **Table S1**: GO-terms associated with karrikin, response to strigolactone, strigolactone metabolic process and strigolactone biosynthetic process.

## Abbreviations

ABA	Abscisic acid
Chr	Chromosome
CRL	Cullin-RING ubiquitin ligase
DLK2	DWARF 14-like2
GA	Gibberellic acid
GO	Gene ontology
GWAS	Genome-wide association studies
IAA	Indole-3-acetic acid
JA	Jasmonic acid
KAI2	Karrikin insensitive 2
KAR	Karrikin
KL	KAI2-ligand
LRR	Leucine-rich repeat containing domain
MAX2	More Auxiliary Branches
NO2	Nitrogen oxide
SCF	Skp-Cullin-F-box
SL	Strigolactone
SMXL	Suppressor of MAX-like
TMB	Trimethylbutanolide
TF	Transcription factor
QTL	Quantitative trait locus
